# The role of FDG-PET/CT in preoperative staging of sentinel lymph node biopsy-positive melanoma patients

**DOI:** 10.1186/s13550-016-0228-1

**Published:** 2016-10-05

**Authors:** Evan C. Frary, Dorte Gad, Lars Bastholt, Søren Hess

**Affiliations:** 1 Department of Nuclear Medicine, Odense University Hospital, Sdr. Boulevard 29, 5000 Odense C, Denmark; 2 Department of Radiology and Nuclear Medicine, Hospital of South West Jutland, Finsengade 10, 6700 Esbjerg, Denmark; 3 Department of Clinical Research, Faculty of Health Sciences, University of Southern Denmark, Wisløvparken 19, 3, 5000 Odense C, Denmark; 4 Department of Plastic Surgery, Odense University Hospital, Sdr. Boulevard 29, 5000 Odense C, Denmark; 5 Department of Oncology, Odense University Hospital, Sdr. Boulevard 29, 5000 Odense C, Denmark

**Keywords:** Skin cancer, Melanoma, Sentinel lymph node biopsy, FDG-PET/CT, Staging

## Abstract

**Background:**

On April 1, 2015, Odense University Hospital (OUH) began a new diagnostic strategy, wherein all malignant melanoma (MM) patients in the Region of Southern Denmark with a positive sentinel lymph node biopsy (SLNB) underwent FDG-PET/CT preoperatively prior to lymph node dissection (LND). The purpose of this study is to determine FDG-PET/CT’s efficacy in finding distant metastasis in the first year after the implementation of this new strategy, and to what extent these findings influence subsequent diagnostic testing and treatment in this patient group. We conducted a retrospective multicenter cohort study which included all patients with MM from all hospitals in the Region of Southern Denmark from April 1, 2015 to April 1, 2016 found to be SLNB-positive who subsequently underwent FDG-PET/CT. Patient information was acquired from the Danish Melanoma Database and was cross-referenced with OUH’s patient records. The data was analyzed for a number of parameters including FDG-PET/CT findings and treatment strategy. Median follow-up time was 7 months.

**Results:**

A total of 47 patients were eligible from the first year of this new diagnostic strategy. One patient was excluded due to undergoing LND prior to FDG-PET/CT. Thus, 46 patients were included in this study. Ultimately, preoperative FDG-PET/CT neither uncovered any distant metastases nor led to any alterations in treatment strategy in this patient group.

**Conclusions:**

Surprisingly, this new diagnostic strategy did not find any MM metastases or uncover anything else of relevance. FDG-PET/CT did, however, provide false positive findings in 13 % (6/46) of these patients. These scans triggered additional, predominantly invasive, procedures, which did not ultimately have an impact on the therapeutic strategy. Thus, these findings indicate a need for re-evaluation of this new diagnostic strategy as well as the necessity for further clinical trials evaluating FDG-PET/CT’s utility in this clinical setting.

## Background

The incidence of malignant melanoma (MM), and its corresponding health burden, has been steadily increasing over the past few decades with 232,000 new cases diagnosed worldwide in 2012 [1]. MM’s mortality rate varies greatly depending on the stage of the disease at diagnosis. In the early stages of MM, the 5-year survival rates are quite high, 97 and 92 % for stages IA and IB, respectively. This survival rate, however, falls drastically in the later stages of MM to between 10 and 30 % for stage IV patients [2]. This vast difference in prognosis underscores the importance of early diagnosis and accurate staging of MM.

Positron emission tomography and computed tomography (PET/CT) based on a radio-labeled glucose analog known to accumulate in hypermetabolic cells (e.g., cancer cells), F-18-fluordeoxyglucose (FDG), is increasingly being employed in the diagnosis, staging, and evaluation of treatment response in a multitude of malignancies [3, 4]. While sentinel lymph node biopsy (SLNB) is still considered the reference standard for determining locoregional lymph node metastasis [5], the role of FDG-PET/CT in the staging of MM patients has yet to be conclusively defined.

A number of papers have suggested that for the early stages of MM (i.e., I-II) FDG-PET/CT is only of limited diagnostic value, due to its low sensitivity in detecting microscopic lymphatic disease [6–8]. It has been posited, however, that in advanced stages of MM (i.e., III-IV), FDG-PET/CT can be of great value by locating distant metastases, thereby influencing treatment decisions and informing prognosis [8–12]. As the primary treatment for stage III patients is radical lymph node dissection (LND) [13], the identification of these distant metastases prior to LND is of great importance as this treatment becomes superfluous in stage IV metastatic patients. Thus, LND, and its corresponding complications, can be avoided if these metastases are found preoperatively, as well as allowing for the swift initiation of subsequent therapeutic strategy.

On April 1, 2015, Odense University Hospital began a new diagnostic strategy, wherein all MM patients in the Region of Southern Denmark with positive SLNB underwent FDG-PET/CT preoperatively prior to LND. In this article, we report on the efficacy of FDG-PET/CT in finding distant metastasis in patients with SLNB-positive MM, as applied in this new diagnostic strategy, and to what extent this imaging modality influences subsequent diagnostic testing and treatment.

## Methods

### Study population

We conducted a retrospective multicenter cohort study which included all MM patients with positive SLNB from all hospitals in the Region of Southern Denmark from April 1, 2015 to April 1, 2016. The data for this study was collected from the Danish Melanoma Database (DMD), which contains information on all MM patients in Denmark, as well as the Region of Southern Denmark’s electronic patient record and imaging system. The inclusion criteria for the study were a novel diagnosis of MM, a positive SLNB for MM, and a subsequent FDG-PET/CT prior to LND. The patients’ medical records were reviewed and cross-referenced with the DMD for basic information comprising age and sex as well as primary tumor characteristics (location, histological type, Clark’s level, Breslow’s depth, and ulceration presence), SLNB findings (location, number of positive sentinel lymph nodes (SLN) removed, and total number of SLN removed), TNM stage, FDG-PET/CT findings, and finally the additional tests and/or changes in treatment triggered due to the FDG-PET/CT findings. Follow-up analysis was performed by reviewing the patients’ electronic records with a median of 7 months (range 3–13).

Due to the retrospective nature of the study, informed consent was not required by Danish Law, but the study was approved by the Danish Data Protection Agency (journal nr. 15/48420) and the Danish Board of Health in accordance with Danish legislation.

### FDG-PET/CT-protocol

All FDG-PET/CT scans were performed routinely in accordance the department’s standard protocol, i.e., with scans from vertex of the skull to the proximal femurs or the entire body if the primary lesion was in the lower extremities. The local protocol is in accordance with guidelines from the European Association of Nuclear Medicine [14]. All examinations were performed on a GE Discovery VCT, a GE Discovery STE, a GE Discovery RX, or Discovery 690 PET/CT scanner (GE Healthcare, Milwaukee, WI, USA). The CT imaging was performed as a low-dose CT scan without contrast enhancement. Data were reconstructed with a standard filter into transaxial slices with a field of view of 50 cm, matrix size of 512 × 512 (pixel size 0.98 mm), and a slice thickness of 3.75 mm. The CT scan was followed immediately by a PET scan performed using a standard whole-body acquisition protocol with six or seven bed positions and an acquisition time of approx. 2.5 min per bed position (adjusted to patient size). The scan field of view was 70 cm. Attenuation correction was performed from the CT scan. The PET data were reconstructed into transaxial slices with a matrix size of 128 × 128 or 256 × 256 (Discovery 690) and a slice thickness of 3.75 mm using iterative 3D OS-EM (with varying iterations and subsets), and displayed in coronal, transverse, and sagittal planes. Corrections for attenuation, randoms, dead time, and normalization were done inside the iterative loop. At the time of FDG administration, all patients had fasted for at least 6 h. PET/CT image acquisition commenced 60 ± 5 min. after the administration of a weight adjusted dose of 4 MBq/kg (110 μCi/kg) FDG (min. 200 MBq (5 mCi) and max. 400 MBq (10 mCi)). Analysis of the PET and fused PET/CT data was done using a GE Advantage Workstation v. 4.4 or a GE Advantage Server 2.0 (GE Healthcare). The fused PET/CT imaging analysis was performed as a routine interpretation by a nuclear medicine specialist.

### Imaging analysis

All FDG-PET/CT findings were confirmed or rejected using histological analysis and/or follow-up as the reference standard. Increased FDG-uptake suggestive of metastasis was defined as a true positive result if subsequently confirmed via histological analysis and false positive if subsequently ruled out by histological analysis or, as in one case, by a pulmonologist. If there was no abnormal FDG-uptake suggestive of metastasis, a true negative result was defined as no metastasis uncovered in the follow-up period and false negative where a metastasis was subsequently discovered in the follow-up period.

### Statistical analysis

FDG-PET/CT’s diagnostic efficacy, and the corresponding 95 % confidence interval, was calculated with respect to sensitivity, specificity, positive predictive value (PPV), negative predictive value (NPV), and accuracy. All statistical analyses were done in STATA/IC 14.0 for Windows.

## Results

Forty-seven consecutive SLNB-positive MM patients were eligible for this study. Due to scheduling conflicts, one patient underwent LND prior to FDG-PET/CT and was therefore not included. Thus, a total of 46 patients, 26 males and 20 females with a median age of 61 (range 26–82), were included in our retrospective patient cohort.

### Primary tumor characteristics

The locations of the patients’ primary tumors were as follows: head and neck (*n* = 1), trunk (*n* = 21), upper extremities (*n* = 7), and lower extremities (*n* = 16). After histological analysis, the diagnoses were: superficial spreading melanoma (*n* = 31), nodular melanoma (*n* = 9), acral lentiginous melanoma (*n* = 3), spitzoid melanoma (*n* = 1), and melanoma of undetermined type (*n* = 1). The vast majority of the patients had a Clark’s level of IV (*n* = 37), the rest had a Clark’s level of either V (*n* = 3), III (*n* = 4), or II (*n* = 1). The Breslow’s depths were divided into four intervals and tallied: ≤1.00 mm (*n* = 2), 1.01–2.00 mm (*n* = 16), 2.01–4.00 mm (*n* = 23), and >4.00 mm (*n* = 4). Histological analysis of the primary tumor also revealed ulceration in 18/45 (40 %) and mitoses in 21/45 (47 %). Note, one patient had two simultaneous cutaneous tumors and the primary tumor could not be conclusively determined; thus, these tumors were not included in the tumor tally. Table 1 provides a description of the patients’ primary tumor characteristics.

**Table 1:** Primary tumor characteristics

Primary tumor characteristics	No. (*n* = 45)^a^
Location	
Head and neck	1
Trunk	21
Upper extremity	7
Lower extremity	16
Melanoma type	
Superficial spreading	31
Nodular	9
Acral lentiginous	3
Spitzoid	1
Undetermined	1
Clark’s level	
I	0
II	1
III	4
IV	37
V	3
Breslow’s thickness	
≤1.00 mm	2
1.01–2.00 mm	16
2.01–4.00 mm	23
>4.00 mm	4
Ulceration present	18
Mitoses present	20

^a^One patient had two simultaneous cutaneous tumors and, as it was unclear as to which was the primary tumor, was not included in this table

### SLNB findings

With respect to the patients’ SLNB, the procedures were performed on lymph nodes located in the axilla (*n* = 25), the inguinal canal (*n* = 24), the neck (*n* = 3), and the popliteal fossa (*n* = 1). All patients had at least one positive SLNB. In total, 120 lymph nodes were removed from the entire cohort and 65 of them (54 %) were determined to be positive for MM metastasis. The mean tumor diameter found for a positive SLN was 0.90 mm. Perinodal growth was seen in three patients. The patients’ preliminary staging, prior to PET/CT evaluation, were found to be stage IIIA (*n* = 24), IIIB (*n* = 18), and IIIC (*n* = 4). With respect to TNM staging, the patients’ T stages were diagnosed as T1 (*n* = 2), T2 (*n* = 20), T3 (*n* = 20), and T4 (*n* = 4), while the N stages were N0 (*n* = 0), N1 (*n* = 32), N2 (*n* = 12), and N3 (*n* = 2). Note, with respect to the aforementioned patient with two simultaneous cutaneous tumors, the tumor with the higher T staging was used. No distant MM metastases were diagnosed; thus, all patients had M0-disease. Table 2 provides a description of the patients’ SLNB findings and preliminary staging as well as final staging.

**Table 2:** SLNB findings, preliminary staging, LND findings, and final staging

SLNB findings, preliminary staging^a^, LND findings, and final staging^b^	
SLNB findings	
SLNB location	
Axillary lymph nodes	24
Inguinal lymph nodes	24
Cervical lymph nodes	3
Popliteal fossa lymph nodes	1
SLN	
Removed	
Total	120
Per patient median (range)	2 (1-5)
Positive	
Total	65
Per patient median (range)	1 (1-5)
SLN tumor diameter mean (mm)	0.90
Perinodal growth	3
Preliminary staging	
IIIA	24
IIIB	18
IIIC	4
LND findings	
NSLN	
Removed	
Total	564
Per patient median (range)	12 (3-45)
Positive	
Total	5
Per patient median (range)	0 (0-1)
Final staging	
IIIA	24
IIIB	18
IIIC	4

^a^Preliminary staging was done post-SLNB and pre-PET/CT

^b^Final staging was done post-LND

*SLNB* sentinel lymph node biopsy, *LND* lymph node dissection, *SLN* sentinel lymph node, *NSLN* non-sentinel lymph node

### PET/CT findings

Increased FDG-uptake suggestive of distant metastasis was found in 7/46 patients (15 %). One of these was determined to be progression of pre-existing concomitant squamous-cell carcinoma of the lungs and not metastatic MM. The remaining 6/46 patients (13 %) had an increased FDG-uptake suggestive of MM metastasis, and all six triggered additional diagnostic testing. Table 3 provides an overview of the positive FDG-PET/CT findings and subsequent tests performed.

**Table 3:** PET/CT findings in patients with increased FDG-uptake suggestive of MM distant metastasis (*n* = 6)

No.	Age/sex	FDG-uptake location(s)	Additional tests	Distant metastasis	Treatment outcome
1	45/M	Axillary LN, colon, rectum	Colonoscopy, polyp resection	No	Lymph node dissection
2	60/F	Lung infiltrates bilaterally	Pulmonologist referral	No	Lymph node dissection
3	54/M	Mediastinum LN, hilar LN	EBUS biopsy	No	Lymph node dissection
4	74/F	Thyroid gland	Ultrasound-guided biopsy	No	Lymph node dissection
5	71/M	Sigmoid colon	Colonoscopy, polyp resection	No	Lymph node dissection
6	74/M	Sigmoid colon	Colonoscopy, polyp resection	No	Lymph node dissection

*MM* malign melanoma, *LN* lymph nodes

**Fig. 1:**
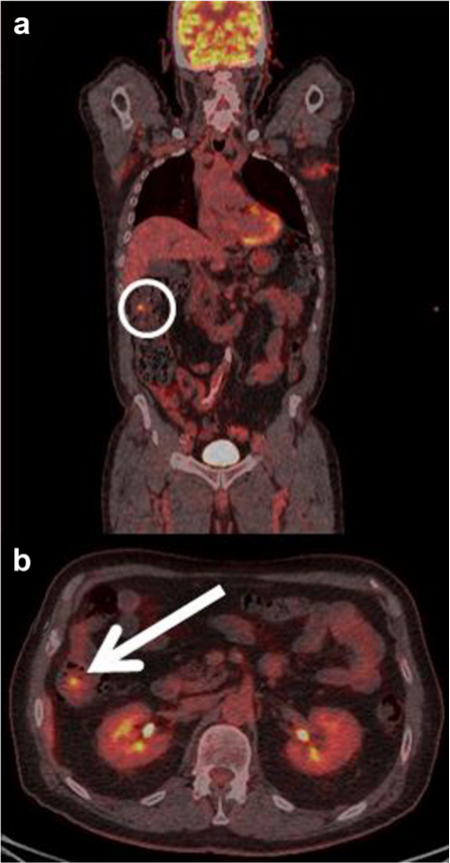
**a** Coronal-fused FDG-PET/CT showing the region of interest (*circle*). **b** Axial-fused FDG-PET/CT showing increased uptake in the colon and rectum (*arrow*)

**Fig. 2:**
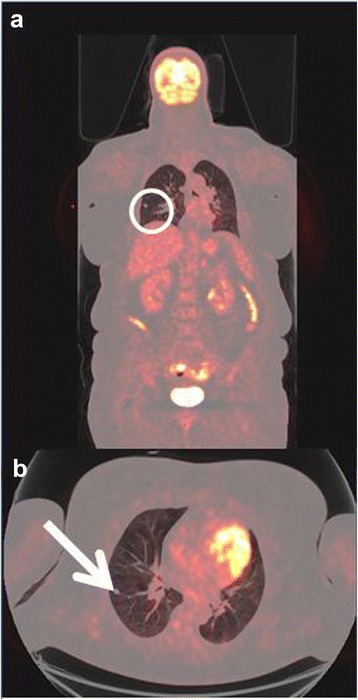
**a** Coronal-fused FDG-PET/CT showing the region of interest (*circle*). **b** Axial-fused FDG-PET/CT showing increased uptake in lung infiltrates (*arrow*)

**Fig. 3:**
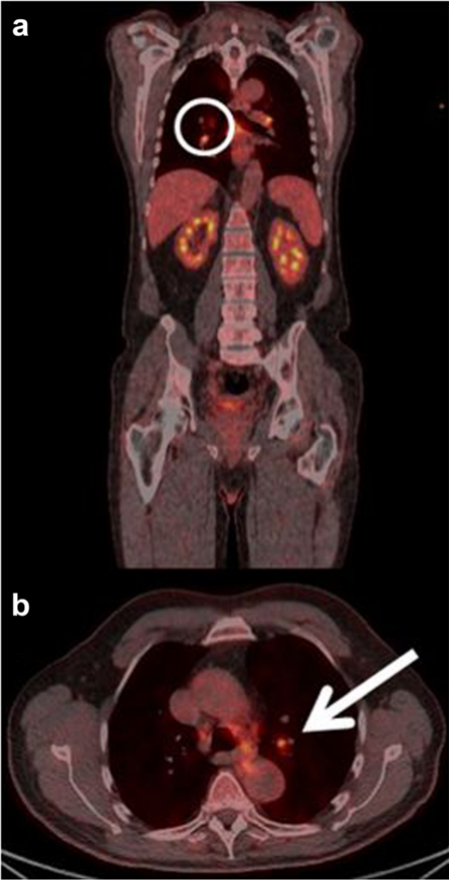
**a** Coronal-fused FDG-PET/CT showing the region of interest (*circle*). **b** Axial-fused FDG-PET/CT showing increased uptake in the hilar lymph nodes (*arrow*)

**Fig. 4:**
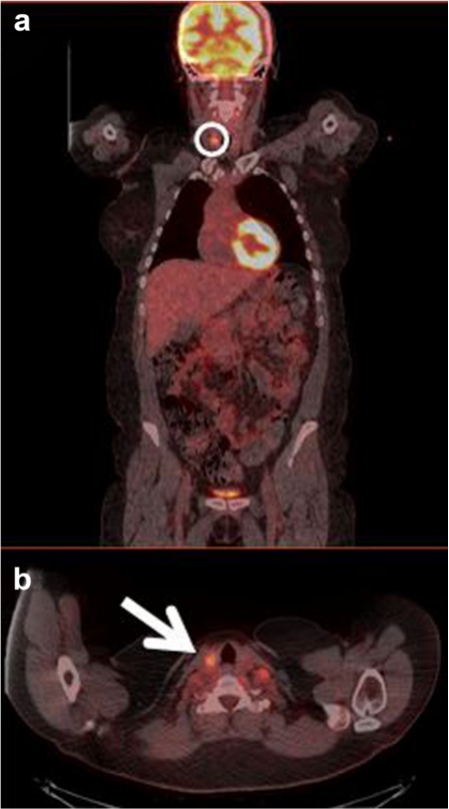
**a** Coronal-fused FDG-PET/CT showing the region of interest (*circle*). **b** Axial-fused FDG-PET/CT showing increased uptake in the thyroid gland (*arrow*)

**Fig. 5:**
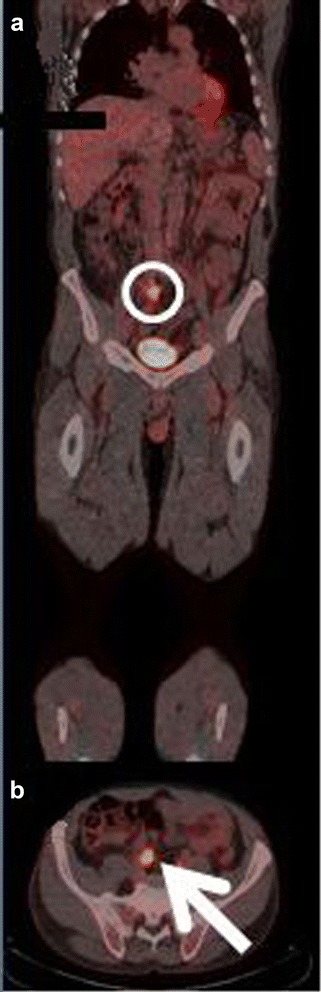
**a** Coronal-fused FDG-PET/CT showing the region of interest (*circle*). **b** Axial-fused FDG-PET/CT showing increased uptake in the sigmoid colon (*arrow*)

**Fig. 6:**
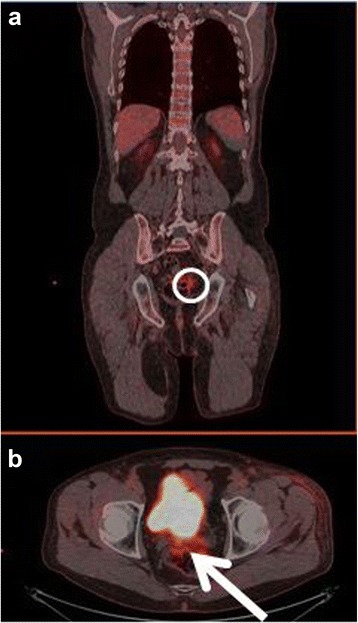
**a** Coronal-fused FDG-PET/CT showing the region of interest (*circle*). **b** Axial-fused FDG-PET/CT showing increased uptake in the sigmoid colon (*arrow*)

The first patient had increased uptake in the axillary lymph nodes as well as in the colon and rectum (Fig. 1) and was referred to a colonoscopy where one benign polyp was resected. Patient number two had bilateral lung infiltrates upon FDG-PET/CT imaging (Fig. 2), and was referred to a lung specialist where no further testing was undertaken. The third patient was also referred to a lung specialist due to increased FDG-uptake in the lymph nodes in the mediastinum and lung hili (Fig. 3). The patient underwent endobronchial ultrasound (EBUS) biopsy of the suspect nodes, and no cancer was found. Patient number four had unilateral increased uptake in the thyroid gland (Fig. 4) and was referred to an ear, nose, and throat (ENT) specialist where an ultrasound-guided biopsy was subsequently performed revealing a benign adenoma. The remaining two patients had increased uptake in the sigmoid colon (Figs. 5 and 6). After referral to a colonoscopy, a polyp was found and determined to be benign in both cases. None of these additional tests ultimately led to any alteration in the patients’ melanoma treatment, and all the patients subsequently underwent LND as planned, except in four instances where the patient refused treatment. Five out of the 42 patients (12 %) who ultimately underwent LND were found to have a positive non-sentinel lymph node (NSLN). The patients’ final staging, i.e., post-LND, remained unchanged from their preliminary staging (i.e., IIIA (*n* = 24), IIIB (*n* = 18), and IIIC (*n* = 4)). Table 2 provides a description of the LND findings and the patients’ final staging.

Ultimately, the analysis of this new FDG-PET/CT diagnostic strategy in this patient group showed 0 true positives (TP), 6 false positives (FP), 0 false negatives (FN), and 40 true negatives (TN). Further statistical evaluation of FDG-PET/CT’s diagnostic probabilities with respect to MM metastasis provided: sensitivity not estimable (due to TP and FN being 0), specificity 87 % (74–95 %), PPV 0 % (CI 0–46 %), NPV 100 % (CI 91–100 %), and accuracy 87 % (CI 74–95 %). Note, while we were able to calculate all the diagnostic probabilities, other than sensitivity, NPV is the only probability of real practical value in this instance.

## Discussion

The results presented above comprise our experience from the first year after the implementation of a new diagnostic strategy of performing preoperative FDG-PET/CT in 46 consecutive SLNB-positive MM patients prior to LND. While 12 % of patients who underwent LND had positive NSLN, similar to the rate (10–17 %) found in the literature [15–17], this novel approach did not find any MM metastases or lead to any alterations in treatment strategy. FDG-PET/CT did, however, provide FP findings in 13 % (6/46) of these patients. This triggered additional, predominantly invasive, procedures, which did not ultimately have an impact on the therapeutic strategy. While this relatively high FP rate in FDG-PET/CT has been seen previously in similar MM patient populations [7, 18], it does not automatically preclude FDG-PET/CT’s utility in these patients. The FP rate must instead be weighed against the potential for overlooking a distant metastasis which can greatly impact patient prognosis and treatment options. However, our results may provide an incentive to re-evaluate of the use of FDG-PET/CT in this patient population.

This diagnostic strategy was originally initiated in response to literature indicating FDG-PET/CT’s value in preoperative screening of SLNB-positive MM patients. A number of studies have shown FDG-PET/CT, in general, is the superior imaging modality in diagnosing distant metastasis in MM patients, compared to other conventional modalities such as ultrasonography, whole-body MRI, CT alone, or PET alone, with a sensitivity, and specificity as high as 97 % [9, 19, 20]. More specifically, the current consensus in the literature also suggests that the timing of FDG-PET/CT scans is important, as it can have the most significant impact on subsequent treatment decisions if undertaken prior to treatment initiation (e.g., before LND) [6, 8, 10–12, 21, 22]. For example, in one of the first studies on FDG-PET/CT’s impact on predominantly later stage (i.e., III-IV) MM patients, Gulec et al. found FDG-PET/CT imaging led to a change in treatment in 49 % (24/49) [21]. Subsequent studies, such as Schule et al. and Bronstein et al., also supported FDG-PET/CT’s utility in later-stage MM patients finding that it led to significant therapeutic alterations in 59 % (31/52) and 12 % (4/33), respectively [11, 22].

However, these aforementioned studies evaluated a different patient population and, thus, may explain the differences as compared to our results. Our study included only stage III MM patients who were scanned shortly after a positive SLNB, whereas Gulec et al., Schule et al., and Bronstein et al. all included advanced MM patients with both stages III and IV [11, 21, 22]. This difference in sample populations is quite important as patients with established distant metastases are much more likely to have additional distant metastases than patients diagnosed only with locoregional metastases.

Additionally, as in our study, a number of more recent articles have also supported a re-evaluation of FDG-PET/CT’s use in primary staging of SLNB-positive MM patients. A systematic review from Shröer-Günther et al. found that, although FDG-PET/CT’s diagnostic accuracy appeared to increase in higher stages of MM, there was no evidence of benefit to the patient found when FDG-PET/CT is used in primary staging [23]. Several other studies have also questioned FDG-PET/CT’s usefulness in routine scanning of SLNB-positive MM patients [18, 24–26]. Two of these studies, Scheier et al. and Wagner et al., both reported on a patient population almost identical to ours involving 46 SLNB-positive MM patients where FDG-PET/CT was utilized in the initial staging to locate distant metastases [18, 24]. Scheier et al. found that 33 % (15/46) of these patients had abnormal uptake suggestive of MM metastasis, but only 7 % (3/46) were ultimately found to have a metastasis which lead to a change in treatment [18]. Wagner et al. found 13 % (6/46) with abnormal uptake, none of which were ultimately confirmed as a MM metastasis [24]. These articles reinforce our findings that FDG-PET/CT may not be beneficial in newly diagnosed asymptomatic SLNB-positive MM patients. Table 4 provides a list of the relevant aforementioned original articles and their findings.

**Table 4:** Relevant original articles

Author	Study design	Included	Patient population	Diagnostic test(s)	Diagnostic evaluation	Findings
*PET/CT Recommended*						
Gulec et al. [21]	Retrospective	49 patients	MM patients 46 stage III/IV 3 stage II (high risk)	FDG-PET/CT vs. CT plus brain MRI	Treatment change in 49 %	FDG-PET/CT better than CT plus brain MRI for determining extent of disease
Bronstein et al. [22]	Prospective	32 patients	MM patients Clinically evident III Oligometastatic IV	FDG-PET/CT	Treatment change in 12 %	FDG-PET/CT of use preoperatively in surgically treatable metastatic melanoma
Schule et al. [11]	Retrospective	52 patients in primary staging group	MM patients stages III-IV	FDG-PET/CT vs. CT alone	Treatment change in 59 %	FDG-PET/CT better than CT alone for primary staging
*PET/CT not recommended*						
Wagner et al. [24]	Retrospective	46 patients	MM patients with positive SLNB	PET/CT	Treatment change in 0 %	PET/CT provides no benefit for this patient group
Scheier et al. [18]	Retrospective	46 patients	MM patients with positive SNLB	PET/CT	Treatment change in 7 %	PET/CT not recommended for asymptomatic MM patients with positive SLNB micrometastasis

*MM* malignant melanoma, *SNLB* sentinel lymph node biopsy

The main strengths of this study are twofold. Firstly, the consecutive multicenter patient cohort is highly representative of the region’s patient population without selection bias. Secondly, FDG-PET/CT showed a very high NPV. While FDG-PET/CT did not lead to any alterations in treatment strategy, it did correctly rule out distant metastases in all patients. This high NPV (i.e., 100 % with CI 91–100 %) is important as it strengthens the prognosis and helps ensure that the subsequent LND is the appropriate treatment.

There were, however, also a number of limitations in our study. One was the scanning protocol itself. The routine scan field was from the vertex of the skull to the proximal femur in patients where the primary lesion was not located in the lower extremities, i.e., in essence only two-thirds of the body is covered. Thus, in these patients, the potential for metastasis distal to the proximal femur is completely ignored, and this may inadvertently increase the number of FN scans. Although previous studies seem to indicate that the inclusion of the lower extremities is of limited benefit in MM patients [27, 28], a scanning protocol which routinely includes the entire body, regardless of primary lesion location, would eliminate this issue entirely. Another matter in our standard MM scan protocol is the use of low-dose non-contrast-enhanced CT only. It is conceivable that contrast-enhanced CT could lead to additional metastatic findings. However, the added value of contrast-enhanced CT is controversial as some studies have indicated an added value due to increased sensitivity in MM [29], while other studies in cancer patients in general have shown no additional clinically significant effect [30, 31].

While FDG-PET/CT correctly ruled out distant metastasis in all the patients with a NPV of 100 % (CI 91–100 %), our study’s relatively short follow-up period, median 7 months (range 3–13), lessens its conclusive power. For example, Wagner et al. found 12 % (5/40) of FDG-PET/CT to be FN after 12 months follow-up [24]. A longer follow-up period would have strengthened the reliability of our study’s high NPV. Finally, the limited number of patients and the retrospective nature of the study, albeit very similar in scope and size as a number of other comparable studies, restrict the conclusions that can be drawn. While a re-evaluation of the current recommended diagnostic strategy is warranted based on the findings from our study, further research in the form of a randomized clinical trial would be of great benefit for a more definitive determination of the true value of FDG-PET/CT in this clinical setting.

## Conclusions

Surprisingly, and contrary to the preponderance of evidence in the literature, this novel diagnostic strategy revealed no distant MM metastases or anything else of relevance during its first year. FDG-PET/CT did, however, trigger a number of additional diagnostic procedures, none of which led to a change in treatment or the diagnosis of any other serious illness. Although our patient population is limited, these findings indicate a need for re-evaluation of the diagnostic strategy including considerations about optimal scan protocol and CT procedure as well as further evaluation in a randomized clinical trial.

## Abbreviations

LND: Lymph node dissection
MM: Malignant melanoma
NPV: Negative predictive value
OUH: Odense University Hospital
PPV: Positive predictive value
SLN: Sentinel lymph node
SLNB: Sentinel lymph node biopsy
